# Modelling Processes and Products in the Cereal Chain

**DOI:** 10.3390/foods10010082

**Published:** 2021-01-04

**Authors:** Otilia Carvalho, Maria N. Charalambides, Ilija Djekić, Christos Athanassiou, Serafim Bakalis, Jose Benedito, Aurelien Briffaz, Cristina Castañé, Guy Della Valle, Isabel Maria Nunes de Sousa, Ferruh Erdogdu, Aberham Hailu Feyissa, Nickolas G. Kavallieratos, Alexandros Koulouris, Milica Pojić, Anabela Raymundo, Jordi Riudavets, Fabrizio Sarghini, Pasquale Trematerra, Alberto Tonda

**Affiliations:** 1 LEAF—Linking Landscape, Environment, Agriculture and Food, Instituto Superior de Agronomia, Universidade de Lisboa, Tapada da Ajuda, 1349-017 Lisboa, Portugal; motiliac@isa.ulisboa.pt (O.C.); isabelsousa@isa.ulisboa.pt (I.M.N.d.S.); anabraymundo@isa.ulisboa.pt (A.R.); 2 Department of Mechanical Engineering, Imperial College London, London SW7 2AZ, UK; m.charalambides@imperial.ac.uk; 3 Faculty of Agriculture, University of Belgrade, 11000 Belgrade, Serbia; 4 Department of Agriculture Crop Production and Rural Environment, University of Thessaly, 38443 Volos, Greece; athanassiou@uth.gr; 5 Department of Food Science, University of Copenhagen, DK-1958 Frederiksberg, Denmark; bakalis@food.ku.dk; 6 Department of Food Technology, Universitat Politècnica de València, Camino de Vera, 46022 Valencia, Spain; jjbenedi@tal.upv.es; 7 UMR QUALISUD, CIRAD, 34398 Montpellier, France; aurelienbriffaz@gmail.com; 8 IRTA, 08348 Cabrils, Spain; cristina.castane@irta.es (C.C.); jordi.riudavets@irta.cat (J.R.); 9 UR 1268 BIA, INRAE, 44316 Nantes, France; guy.della-valle@inrae.fr; 10 Department of Food Engineering, Ankara University, 06830 Ankara, Turkey; ferruherdogdu@ankara.edu.tr; 11 Production Engineering, National Food Institute, Technical University of Denmark (DTU), 2800 Kongens Lyngby, Denmark; abhfe@food.dtu.dk; 12 Faculty of Crop Science, Agricultural University of Athens, 11855 Athens, Greece; nick_kaval@aua.gr; 13 Department of Food Science and Technology, International Hellenic University, 57001 Thessaloniki, Greece; akoul@food.teithe.gr; 14 Institute of Food Technology, University of Novi Sad, 21000 Novi Sad, Serbia; milica.pojic@fins.uns.ac.rs; 15 Department of Agriculture, University of Naples Federico II, 80138 Napoli, Italy; sarghini@unina.it; 16 Department of Agriculture, Environment and Food Sciences, University of Molise, 86100 Campobasso, Italy; trema@unimol.it; 17 UMR 518 MIA, INRAE, Paris-Saclay University, 75005 Paris, France

**Keywords:** cereals, food transformation, modelling, transformation processes

## Abstract

In recent years, modelling techniques have become more frequently adopted in the field of food processing, especially for cereal-based products, which are among the most consumed foods in the world. Predictive models and simulations make it possible to explore new approaches and optimize proceedings, potentially helping companies reduce costs and limit carbon emissions. Nevertheless, as the different phases of the food processing chain are highly specialized, advances in modelling are often unknown outside of a single domain, and models rarely take into account more than one step. This paper introduces the first high-level overview of modelling techniques employed in different parts of the cereal supply chain, from farming to storage, from drying to milling, from processing to consumption. This review, issued from a networking project including researchers from over 30 different countries, aims at presenting the current state of the art in each domain, showing common trends and synergies, to finally suggest promising future venues for research.

## 1. Introduction

The food supply chain is usually considered as a system including organizations, people, activities, information, and resources that interact with each other for the purpose of moving food or a food service from suppliers to consumers [1]. The complexity in the food chain depends on several factors, such as the number of links within the chain, the number of processing steps, the number of raw materials, the number of suppliers of raw materials, the logistics and the destination of products, influencing the necessity of a higher level of control [2]. Within the food chain, several criteria such as environmental, hygiene, economical, functional, health and quality play significant roles, where different stakeholders, with divergent goals raise different arbitration issues [3]. In the scope of this work, the cereal chain consists of production of various types of cereal grain (wheat, maize, barley, rice, etc. production of intermediate/final products (flour, bread, biscuits, pasta, etc.), transportation and storage activities, retail and cereals purchasing and consumer patterns [4].

**Figure 1:**
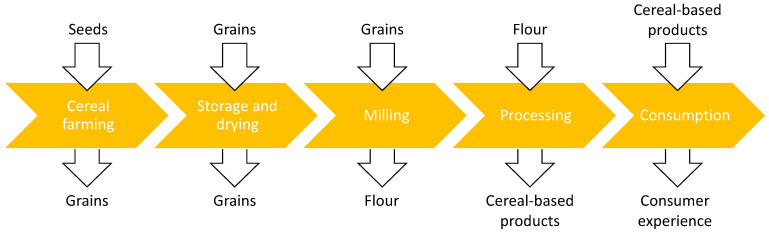
High-level scheme, featuring the stages of the cereal chain.

Modelling of food products and food processes is a complex task, due to several constraints such as lack of knowledge concerning mechanisms that occur within food products and/or food processes, the difficulty of obtaining reliable data, and the uncertainties inherent to food properties [5]. Research on modelling in the food chain includes models related to food products, processes, and systems in which food companies operate, as outlined for example by an environmental review on food modelling [6]. This *product-process-system* approach enables the understanding of food models of any food supply chain, since modelling captures relevant characteristics and features of a phenomenon in complex food products/processes (in a given context), based on theoretical knowledge and experimental measurements. Therefore, to thoroughly understand a supply chain it is important to analyze it using different types of models, from different perspectives (bottom-up/ top-down), and with different scaling tools, to enable an exhaustive overview of its complexity. A generic Input-Process-Output (IPO) model of the cereal chain is presented in Figure 1 depicting main processes and products’ transformation flow, from seeds to final cereal-based products. It clearly identifies five links in the cereal supply chain: farms, storage areas, mills, food processing plants, and consumers as final actors in the supply chain pipeline. Within each process stage of the supply chain continuum, main inputs and outputs are recognized as follows: (i) seeds transformed to grains as a result of cereals farming; (ii) grains stored and preserved for further processing in the storage/drying stage; (iii) grains transformed to flour in mills; (iv) flour processed into cereal-based products within food processing plants; and (v) consumer experience perceived from consuming cereal-based products. The rationale for choosing this generic scheme was to outline possible modelling perspectives in the cereal chain.

The objective of this review is to present main research streams for analyzing food models in the cereal chain, and identify promising future research perspectives. For this particular review we focus exclusively on mathematical and statistical models, that is, representations of systems that can simulate possible outcomes of processes, or representations of products that attempt to predict their properties, both of which can be run on general-purpose software systems. When tackling the cereal chain, we decided to exclude the modelling of logistics, for example the transport of intermediate products from one processing facility to the next, as there the specialized literature is substantial, and mostly offers optimization solutions that are independent from the type of product shipped. Our world’s future relies on finding solutions to two immense challenges: Climate change and the resurgence of pandemics, as made evident by the COVID-19 crisis. Reducing current high levels of food waste in consumers’ homes, as well as in production, is urgently needed to contribute towards the zero-pollution goal and to mitigate climate change. Furthermore, the current COVID-19 pandemic affected the food sector leading to new production and distribution challenges. To mitigate negative impacts of the pandemic on distribution and sales of food, several governments implemented important changes: for example, the Chinese government launched platforms to support food supply [7]. The latest multi-country survey study revealed that the level of maturity of a food safety system was directly correlated with their responses to the challenges raised by the pandemic [8]. This survey also identified food retailers as the weakest link in the food supply chain, the mostly affected by the pandemic. This disrupted the prevailing efficient and highly streamlined ‘just-in-time’ supply chain. In order to deal with such complex problems related to the food chain, modelling of the various links of the chain, as well as the whole chain, is urgently needed, to plan efficient optimizations, flexibility, and resilience in our food systems.

## 2. Modelling Cereal Farming

Given the importance of cereal products as a primary source of food for human population, it is not surprising that modelling of cereal farming in literature is mostly focused on predicting crop yields and the impact of the crops on soil. As the amount of available data on crop production increases, a considerable number of data-driven approaches have been proposed, see [9] for a recent review on the subject. More classical models, such as Crop Estimation through Resource and Environment Synthesis (CERES) [10], Erosion-Productivity Impact Calculator (EPIC) [11] or Model for Nitrogen and Carbon dynamics in Agroecosystems (MONICA) [12], rely upon systems of differential equations instead. Mathematical modelling of cereal harvesting procedures is less explored in literature, with a few notable exceptions [13,14]. Nevertheless, with the growing interest in precision agriculture, and unmanned farming vehicles becoming accessible in the near future, it is predictable that research in harvesting will become more prominent.

## 3. Modelling Storage

Safe storage of grain in silos and warehouses is a complex process, as it could lead to losses of products due to environmental conditions or pests.

Pest control is an extremely important part of storage. Insects found in storage facilities for cereals can present different behaviors and peculiarities, so it is not surprising that models to simulate such pests are often tailored to a restricted number of species and a specific type of product.

The first computer model on stored-products insects was developed by [15] for *Tribolium castaneum* (Herbst) (Coleoptera, Tenebrionidae). This was a single-species, stochastic model that could simulate population development at a specific temperature and relative humidity, and included stage-specific rates of development and survival of the immature stages of four strains.

Reference [16] developed a model for *Ephestia cautella* (Walker) (Lepidoptera, Pyralidae). The model included the amount of food available at a given time, the rate of food ingestion by larvae, mass change of larvae, and the physiological age of the insects.

In [17], a model was developed for simulating energy transfer in wheat stored at 30 °C and 13.5% moisture content, previously infested by *Cryptolestes ferrugineus Stephens* (Coleoptera, Laemophloeidae). The model included rates of insect development and survival, fecundity, respiration, egestion and biomass changes.

The first attempt to develop a model for a stored-product pest that could be used on real grain storage facilities was developed by [18,19] for *Sitophilus oryzae* (L.) (Coleoptera, Curculionidae). The model included effects of temperature and grain moisture on immature development rate, mortality, oviposition, feeding, oxygen consumption and production of frass, water and carbon dioxide. Longstaff and other authors continued developing and validating other models related with this model.

Reference [20] developed a general model for predicting numbers of the lesser grain borer *Rhyzoperta dominica (F.)* (Coleoptera, Bostrychidae) in wheat stores throughout USA. They determined the proportions of wheat stored on-farm or in elevators throughout the year, and the percentage of wheat that is harvested during summer.

Reference [21] developed management models for insect pests of stored wheat, that simulate the effects of temperature and moisture content on the population growth of *T. castaneum*, *C. ferrugineus* and *R. dominica*. Reference [22] expanded this model by including subroutines for aeration, fumigation, and insecticides. In [23], similar models were developed and validated for *Oryzaephilus surinamensis* (L.) *(Coleoptera, Silvanidae*) and *S. oryzae* in wheat. In a comparable fashion, [24] developed a model to simulate the population dynamics of *Cryptolestes pusillus* (Schönherr) (Coleoptera, Laemophloeidae).

Simulating the populations of pests affecting storage facilities makes it possible to develop expert systems that help storage experts take decisions and optimize grain storage. Improvements in decision-making should lead to a greater application of effective storage procedures and thus to reductions in losses [25].

Simulation models that take into account biotic (grain, insect, mites, and microflora) and abiotic (temperature, moisture content, relative humidity, solar radiation, gas composition, etc.) factors can be used to solve various problems in stored-grain management such as the maximum safe storage period of grain, the risk of pest outbreaks, the efficacy of air ventilation, and the degradation of pesticides, etc. [26]. The development of such simulation models requires an effective interaction among several experts such as biologists, biochemists, toxicologists, agricultural engineers, economists, and computer modellers [27].

There are several success stories of decision system software developed by both public institutions and private companies. *Stored Grain Advisor* (https://www.ars.usda.gov/plains-area/mhk/cgahr/spieru/docs/stored-grain-advisor-expert-systems/) [28], for example, is the first of its kind, released by the U.S. Department of Agriculture. The software predicts the likelihood of insect infestations, recommending preventive and remedial actions. It also provides advice on how to sample and identify insect pests of stored wheat.

The *Grain Storage Information System* [29], jointly developed by several Canadian public institutions and research centers, aims to transfer improved management strategies to farmers and grain storage managers.

More recently, the Grain Research and Development Corporation, co-funded by the Australian Government, developed a mobile phone app to assist growers in managing on-farm grain storage, *GRDC storedgrain* (https://grdc.com.au/resources-and-publications/apps/grdc-storedgrain). The app makes it possible to perform on-line recording of storage details and monitoring data for quality assurance tracking.

In the European Union, storage specialists have validated a prototype of an expert system, *QualiS* [30], in pilot-scale experiments in Denmark and the UK. *QualiS* includes four modules: assessment of the initial quality and condition of the grain; planning the optimal storage technical route; monitoring grain condition during storage; and re-planning the storage technical route if the grain condition drifts out of safe storage conditions.

Chinese experts of modelling proposed *QPais* [31], a web-based expert system for assisted identification of stored insect pest species. The expert system can identify 150 different pest species and provide detailed information for dealing with each one. The expert system claims an 85% accuracy rate of correct identification.

Private companies released decision support systems as well: OPI Insector (http://www.advancedgrainmanagement.com/), for example, promises to automate grain sampling with electronic grain probe traps and data analysis. Reference [32] used this procedure to develop statistical models to predict insect density, and later integrated the workflow they designed inside Stored Grain Advisor.

## 4. Modelling Drying

After the harvest, in order to maintain cereal crops in prime condition over a long storage period, the application of post-harvest drying procedures is often necessary [33]. The drying of cereals inhibits the growth of bacteria, yeasts, and mold, and the development of insects and mites. Through drying, moisture in grains is reduced from values around 17–30% *w*/*w* to 8–15% *w*/*w* [34].

Temperature and moisture distribution inside dried products are major concerns in a drying problem, and the availability of reliable mathematical models is required to properly design the process. Hot air drying is normally used for cereal grains. The drying capacity depends on air temperature, relative humidity, and grain moisture. Maximum drying temperatures range from 43 to 80 °C, depending on the type of cereal and its final application. When designing a hot air drying process, it is important to reduce energy consumption while preserving product quality. In this regard, model-based optimization makes it possible to explicitly formulate both energy and quality objectives, and it has therefore a large potential in enhancing drying process design [35]. In this regard, the modelling of grain drying is focused on thin-layer drying under controlled conditions of air temperature, relative humidity and air velocity, mainly with the aim of assessing the optimal temperature for production of quality dried grains. However, in deep-bed drying of agricultural crops, the conditions of grain and air change with position and time. Consequently, deep-bed drying can be modelled as being composed of a series of several thin layers, where each layer modifies the quality of the air [36].

Considering the inefficiency of convective air drying for several food products, infrared heating could represent an alternative innovative technology: References [37] and [38] reported a detailed review on the fundamentals of infrared heating, and its possible applications for use in drying process of the food products.

Another alternative method presenting advantages over conventional drying techniques is intermittent drying. This technology is based on the implementation of varying-time conditions such as temperature, air rate, and chamber pressure. This drying technique offers better energy efficiency than conventional drying technologies, due to a reduced heat input, a shorter heating time, and a lower total air flow. In addition, product quality may be improved as a result of lower average material temperature and shorter exposure time to hot air. On the other hand, there are more parameters to tune. Reference [39] proposed an enthalpy-drive optimization of the parameters of this process, using a diffusion model. Analyzing the impact of intermittent drying on agricultural products, [40] employed a mechanistic model to find lower surface temperatures and drying times, when compared to continuous hot-air drying.

Microwave drying could represent another viable alternative. While studies on the effects of microwaves on cereal can be found in literature [41], along with mathematical modelling of microwave drying on other agricultural products [42], for cereals only a few predictive models of microwave drying are available [43,44].

Another promising technique is ultrasonic-assisted drying, that boasts the ability to help remove moisture without significantly heating the product by accelerating the mass transfer processes, as confirmed by Finite Element Method (FEM) models [45]. Further studies on the same methodology delivered models providing evidence that ultrasounds may affect both external and internal resistance to mass transfer during drying of cereal grains [46,47]. Modelling cracks during drying of foods using Finite Element Analysis (FEA) is possible through the use of cohesive zone models [48] or, in the case of foods which may be considered as elastic-plastic, progressive damage models [49]. These are complex models which require initial calibration of relevant input parameters such as fracture toughness and critical strain values for damage onset and propagation. Using FEM-based models often leads to problems with numerical convergence due to the large deformations involved for compliant systems such as foods. These can be overcome through the use of the Coupled Eulerian – Lagrangian Finite Elements Analysis [50] or alternative ‘meshless’ methods such as the Discrete Element [51,52] or the Smoothed Particle Hydrodynamics [53,54] methods.

Finally, near-infrared spectroscopy (NIRS) showed considerable promise in analyzing the results of drying. Modelling studies based on univariate and multivariate approaches [55] validated the NIRS approach, and subsequent works provided a model for chemometric optimization of this technique [56].

## 5. Modelling Milling

After the drying step, cereal grains are usually processed in mills, obtaining flour that is later used for further processing. Milling operations can gain efficiency by using Discrete Element Modelling (DEM) to understand how energy is dissipated, to create and propagate cracks at the different levels of grain organization. Indeed, when grains come in contact, forces are transmitted through interfaces in the grinders. While this approach requires massive parallel computing, it can predict the variations of distribution in size of particles with grain microstructure and mechanical loading, showing that structural defects (microporosity, craze, wall discontinuity) are the most likely paths for crack propagation, and that the rupture stress of the grain increases as the grain size decreases [57].

**Figure 2:**
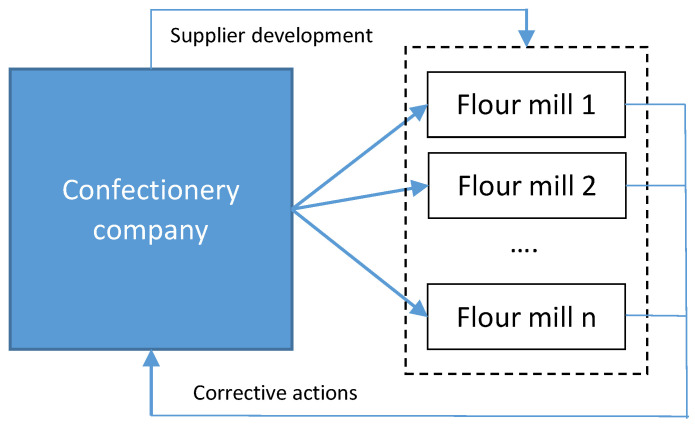
Scheme of supplier evaluation for confectionery.

Models have also been developed for evaluating flour-mills suppliers in the confectionary supply chain, using second-party audits [58], see Figure 2. The figure outlines the scheme used for evaluating suppliers (mills) organized and performed by the largest confectionery company in Serbia. The model had two main purposes: to assess quality and food safety systems of mills as suppliers, and to evaluate the level of development of their systems. During the first assessment, various nonconformities were identified, and suppliers were asked to raise and implement corrective actions. Within the first round, average effectiveness score was 38.6 (out of 100). After 12 months, effectiveness of implemented measures was re-evaluated and suppliers achieved 64.4 (out of 100), verifying improvements of their quality and food safety systems. However, this research confirmed that the certification status of a quality and food safety system does not necessarily imply a high performance. Also, all companies in the supply chain experienced problems in identifying their processes, setting key performance indicators/objectives for evaluating effectiveness of their processes, and had no problem solving tools in place. Within quality/food safety control, the majority of companies do not document their control methods, and have no procedures in place to verify consistency in their results. Control of hazards and risks in terms of food safety and HACCP implementation was identified as the main food safety constraint. Control is one of the most important processes in the food industry, that quality and food safety systems both strongly rely on [59].

## 6. Modelling Processing

Once flour is obtained, different types of processing can be used, to obtain a variety of cereal-based products.

### 6.1. Wheat Flour Mixing

During wheat flour mixing, Specific Mechanical Energy (SME) governs the transition from a divided solid medium (the flour) into a deformable continuous one, the flour dough, as it reflects gluten network structuring in the dough. The non-Newtonian viscoelastic behavior of dough has been addressed by a 2D numerical approach, but the continuous changes of its free surface due to the motions of the mixing arm and bowl have not been taken into account yet [60]. A qualitative model has been developed in order to formalize expert’s knowledge and then implemented in a Knowledge Base System (KBS) that makes it possible to compute these sensory criteria as a function of process parameters (input variables). Whilst being based on conventional modelling approach, this KBS can be used by professionals in order to simulate, for instance, the effects of ingredients and mixing parameters on dough consistency, and other criteria the professional baker uses to estimate quality [61]. Models for bread making combining the scientific understanding of material changes, reflected by variations of rheological properties, are called basic knowledge models (BKM). For instance, BKMs relate mixing efficiency to dough proofing capacity, and can be ascertained by a good knowledge of elongational rheology represented by a double power law [62].

### 6.2. Forming Processes and Extrusion

Dough gets processed on a large scale in the food industry and operations such as sheeting and extrusion are very common. Predictive computational models that can be used to determine the level of stresses and strains in the processed food, without expensive trial-and- error physical trials, can function as process optimisation design tools for the food industry. For example, in extrusion, high pressures can potentially lead to significant shearing and tearing, which may alter the microstructure of the food, leading to undesired effects on the quality of the end product. Modelling studies on rolling and extrusion of dough are given in [63,64] which take into account the complex viscoelastic behaviour of the dough material. In addition, micromechanical models have been developed treating dough as a particulate composite consisting of starch particles dispersed in a continuous protein matrix. The effect of the adhesion between the matrix and the filler on the overall mechanical response has been quantified. This is important, as processing has an effect on the microstructure of the dough. Therefore, having models which take into account the specific microstructure can aid in designing processes and products [65].

Pasta production, for example, is considered to be a mature technological process, considering markets acceptance and the widespread use of the final product. Nonetheless, several problems are still present in industrial production, as non-uniform pressure distribution generates uneven extrusion velocity, resulting in different length of extruded product and quality differences in the same production batch. A limited number of works investigated the general rheological properties of pasta dough, with the objective to obtain a better understanding of the basic rheology or mixing behavior [66,67], ultimately proving that pasta dough is a viscoelastic system. Further works showed that, from a computational point of view, the system can be restricted to a simpler purely viscous behavior, and elastic effects can be neglected because shearing flows are dominant through the screw channel and the die [68,69].

Likely because of similarity to polymer processing, FEM numerical modelling of extrusion has drawn some attention in the domain of cereal food processing, either for simulating the influence of extrusion variables or predicting mixing abilities [70]. Nevertheless, the complicated geometry of (twin screw) extruders and the lack of knowledge on the rheological behavior of cereal foods challenge its application. As for wheat flour mixing, during extrusion-cooking of starchy based snacks, SME still governs the transition from a divided solid medium (the flour) into a viscous fluid, the starchy melt, as it reflects starch destructuring (melting and depolymerization during extrusion). Software such as Ludovic^®^ (INRAE/CMREF, Nantes, France) have been designed to predict this variable according to extrusion parameters, making it possible to induce the structural changes of starch [71]. The expansion of the starch melt at the die can be addressed by a phenomenological model that makes it possible to predict the density of the extruded piece, and under reasonable assumptions, its cellular structure [72].

### 6.3. Baking

Contact baking is a process widely used for the production of chapatti, crisp bread, pizzas, grissini, pita bread, etc. Given the long history of this process, its optimization and control to achieve a desired quality are mainly based on human expertise and craftsmanship, in lieu of computational models and engineering computations [73]. In order to apply this latter kind of predictive models, it is necessary to reach a better mechanistic understanding of the contact baking process, including physical phenomena such as heat and mass transfer. This kind of mechanistic mathematical model designed for contact baking needs to consider all phenomena that contribute to the explanation of the physical behavior of the product during the process, in order to better modify the system and ultimately optimize the process.

All the physical and chemical changes in the process are a function of temperature change, affected in turn by simultaneous heat and mass transfer. The evaporation induced by internal porosity together with bread deformation is the major modelling challenge [74,75]. Considering the simultaneous process of heat and mass transfer, experimental determination of the parameters of such transformations constitutes an additional difficulty. The literature mainly focuses on the assumption of an infinite mass transfer coefficient [76,77]. Conversely, the heat transfer coefficient is typically computed using a lumped system method with a high thermal conductivity casting (like aluminium or copper) in the geometry of the baked product. This method apparently loses the effect of surface evaporation, and use of the original process conditions with the baked product itself is suggested for a proper determination of heat transfer coefficient to better evaluate the system parameters in a developed mathematical model [78]. The resulting uncertainty in the modelling of heat and mass transfer in such systems is thus mainly associated with: (1) model assumptions (assumptions made on the underlying physics, to simplify the model), and (2) the values of the parameters.

### 6.4. Rice Cooking

After maize, rice is the second-most consumed cereal in the world. Rice is cooked both by the end users, with domestic appliances, and at the industrial level, with large machines that prepare, for example, convenience rice. The behavior of the rice during cooking and the final perceived quality are both impacted by the modalities of cooking. Rice can be cooked by boiling it in excess water, a method common in Western homes and at the industrial level; or cooked by absorption (so-called Pilaf method), an approach frequently observed in Asia, through electric rice cookers. Characteristics of the cooked rice, for example texture, strongly depend on soaking-cooking conditions like water-to-rice ratio, time, and temperature; but also on properties of the grains, such as geometry and composition. All these parameters directly influence the spatial distribution of several physico-chemical variables, all critical for the quality of the final product, as reported by a considerable amount of works in literature [79,80,81].

For example, soaking-cooking conditions drive the spatial distribution of water content and starch gelatinization of rice grains in time, as can be observed in experiments with Nuclear Magnetic Resonance or Magnetic Resonance Imaging [82,83]. Controlling the texture of the cooked rice requires developing mechanistic modelling methods that make it possible to characterize and predict the physico-chemical transport and reactions of rice grains, while they are being processed. To properly predict the final quality of the texture, several complex phenomena must be concurrently studied: Heat transfer, starch gelatinization, water transport related to grain structure, solid losses, and leaching of starch.

Each step and parameter of the different techniques for cooking rice can have a dramatic impact on the quality of the final product. While it is possible to experimentally observe correlations between specific treatment conditions and variations in texture or sensory properties, the mechanistic relation that goes from processing to kinetics, from molecular alterations to the resulting quality, is often still unclear. Rice grain water uptake kinetics models proposed in literature can be roughly separated into two categories, empirical and mechanistic.

While empirical models can often provide good predictions [84], they do not consider any mechanistic information of water transport processes, and as a consequence their results are often valid for a restricted range of conditions, only. For example, the *linear driving force* is an empirical model able to predict rice grain water uptake for temperatures raising from 25 to 80 °C [85].

On the other hand, mechanistic models try to take into account the underlying physics of a system under study. For soaking-cooking of rice grains in water, the driving force for water mobility is assumed to be the molecular diffusion of water (*D*, measured in m^2^/s, is the apparent water diffusivity), that can be expressed by Fick’s laws, and the matrix is often assumed to be continuous, presenting no porosity. Starting from these ideas, [86] proposed a mechanistic model of a milled long rice grain as a sphere, fitting values of *D* ranging from 2.1 × 10^−10^ m^2^/s to 4.7 × 10^−10^ m^2^/s, with temperature raising from 25 °C to 65 °C.

Another mechanistic approach considered the outer layer of rice (a pericarp, common for paddy or brown rice) as a resistance to mass transfer [87]. Other studies included this resistance in the modelling of *D*; for example, [88] showed that the *D* for a commercial long grain brown rice soaked at 25 °C was nearly three times lower that the *D* of the milled rice.

Soaking can cause hygroscopic stress in rice grains, leading to cracks on the surface, with important consequences on the grain’s water absorption properties. Considering this, [89] proposed a model coupling the mechanical behavior of grains during soaking, hygroscopic swelling, and water transport.

Starch gelatinization in rice can be modeled as a function of temperature and water content. The phase diagram of a Korean round rice variety was presented by [87], while [90] modeled this phenomenon as a first-order reaction, with the quantity of starch reacting at the boundary being proportional to the available surface area of the ungelatinized core. A simulation of rice cooking with Fickian diffusion showed that the effect of swelling is not significant [91].

Despite the considerable advances in mechanical models, an important challenge left to tackle is for the models to take into account, at the same time, the diffusion of water and the thermal transition of starch in rice grains, as these two phenomena seem to be strongly intertwined. The experimental data reported by [92] provide evidence that the water transfer rate dramatically increases when the temperature exceeds the gelatinization point, around 75 °C. Other evidence of the relationship comes from the equilibrium water content, as native starch in the rice grain can only contain a limited amount of water, around 0.5 kg/kg, while the completely gelatinized starch can absorb an amount of water equal to nine times its original dry weight [93]. A possible integration of the two phenomena is presented in [94], where the *water demand* driving force is introduced. The water demand depends on a local maximum water content, which in turn is a function of the local degree of starch gelatinization. Another approach proposed the concept of *two water populations* [87], where one water population is assigned to native starch, with equilibrium water content of 0.5 kg/kg, and a second water population is attached to to gelatinized starch, with a higher equilibrium water content dependant on temperature. This last model also takes into account the local deformation of the rice grains caused by the water uptake. Other models used a spherical rice grain to characterize the position of the gelatinization front based on the water transport equation [95], and a formulation based on the chemical potential to predict both hydration and cooking rates [96].

An important application of in-silico simulation of rice cooking is the optimization of the desired characteristics of the final product, for example texture. Several soaking-cooking scenarios featuring limited amount of waters have been simulated in [97], predicting profiles for both water content and starch gelatinization. The results make it possible to reveal key clues to achieve optimal values of firmness and stickiness in cooked rice. Reference [80] also exploited simulations to evaluate firmness, measuring the force needed to compress the rice grain to a thickness of 0.3 mm, implying a deformation of more than 90% of its initial shape. Such force is heavily correlated with the perceived firmness of the rice.

Other factors that can be taken into account by rice cooking models include the amylose leaching and surface erosion: the amount of amylose solubilized during the process, the amylose-to-amulopectin ratio in the solubilized matter, and the same ratio on the surface of the grains have been all linked to the stickiness of rice [98]. Solid losses in boiling water have been modeled as a power law in function of cooking duration, for ten different rice varieties [99]. Another model, based on molecular diffusion, provided satisfying predictions of soluble amylose diffusion and insoluble solid-phase surface erosion of milled round rice grains [100]. A Fickian diffusion model was also used to predict the concentration of phenolics in rice, relying on the chemical potential [101]. All these results greatly contributed to the understanding and adjustment of stickiness in cooked rice according to consumer demand.

A complete rice cooker was modeled by [102], with the objective of simulating variations in behavior according to different energy profiles, as saving energy is an important factor in food processing, along with the final quality of the products.

Further research is necessary to investigate how each processing step affects the structural, physicochemical and mechanical properties of rice, that ultimately lead to eating quality and sensory perception such as appearance, texture and flavor. In this goal, the use of modelling approaches that are able to predict the physico-chemical changes occurring in the grain during cooking (water transfer, gelatinization, amylose leaching, and surface erosion) could be a bridge between the consumers’ demand of quality and the physico-chemical characteristics of raw grains, and could renew the knowledge about the eating quality of rice.

## 7. Modelling Properties of Cereal Products and Characteristics of Consumption

Due to their porous structure, many cereal foods can be envisioned as solid foams. At the microscopic level, the walls of their cellular structure may be considered as a composite material, a blend of starch and proteins, more or less transformed, containing also polysaccharides (cellulose, hemicellulose, etc.) from the grain blended with water, sugar, and fat. Hence, the texture of these foods, envisioned first as mechanical properties, can be predicted using FEA, provided their organization at different structural levels have been determined and the intrinsic mechanical properties of the components have been measured separately [103].

Nowadays, a considerable attention is focused on the development of products with health claims on the label, which can result in added value for the producer companies. As an example, cookies are popular staple foods in the human diet in many countries, and are generally well accepted by consumers due to their sensory attributes, long shelf life, and convenience. The incorporation of solid components on the biscuit dough, such as dietary fiber, could have serious implications on its structure and perceived texture, which explains the technological limitations for the fiber incorporation. In [104], the authors aim to develop an enriched functional biscuit with Psyllium fiber, and understand the impact of the new ingredient on physicochemical and sensorial properties of the dough and biscuits, using a statistical model.

Several products rely on cereal-based starch as a thickener, stabilizer, or gelling agent, which greatly affects food structure. A fundamental knowledge of the role of starch in large deformation and fracture properties is therefore essential for the development of the desired mechanical properties of a food system. The properties of starch gels have been determined as a function of the recipe (starch concentration), using a high-throughput screening method where the properties of the gels were characterized inversely through probe indentation tests. High throughput screening tests make it possible to automatically test large numbers of different product formulations/recipes, using high speed robotic indenters on small samples moving on conveyor belts. In addition, a wire cutting process was used to determine the fracture properties of the starch gels [105]. More recently, the effect of the mechanical test rate on gelatin gels has been studied, and poroelastic models were used to explain the higher fracture stresses and strains observed as the test rate increases. The gels were treated as a two-phase system with a solid network in a liquid (water) phase. Predictive models were derived, which explained this peculiar fracture behaviour, linking the latter to the microstructural features of the gels, such as the pore size of the network [106].

**Figure 3:**
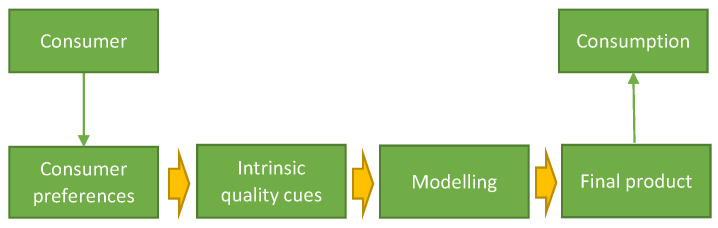
Sequence of steps often used for modelling the effect of consumer preferences in the final product.

The mechanical behaviour of confectionery snacks like wafers is of interest in optimising industrial processes such as cutting, as well as in oral processing, as the latter affects consumer perception of the product. Such products are often cellular foams, and their behaviour will be affected by both the material that constitutes the cell wall (a material effect) as well as the actual microstructure of the cellular arrangement (a geometrical effect). Changing one without changing the other is neither possible, nor cost-efficient. Therefore, predictive models (computational and analytical) are developed and used to understand, optimise and therefore enhance such products. This research can also be used to correlate the mechanical response and sensory perception of confectionery products [63]. In addition, the first bite has recently been modelled with high accuracy using starch-based food products. The structure breakdown and the loads applied on the teeth were obtained and validated with experimental measurements [107,108]. The fracture parameters that are needed as inputs to the simulation of the first bite were derived using novel experimental techniques [109]. From a consumption point of view, it is obvious that various intrinsic quality characteristics prevail in food quality analysis. These intrinsic characteristics in general include taste and food composition [110], such as dietary fibers and enrichment of cereal-based products with various healthy compounds, textural properties and role of starch in food structuring, and food breakdown important in oral processing of food. Figure 3 depicts the sequence of steps used, starting from understanding consumer preferences, transferring their quality cues to models, and finally producing new developed products. It is obvious that we need to understand consumer preferences associated with taste and textural characteristics of cereal-based products, such as wafers or biscuits, and better characterize phenomena occurring in the products, to enable successful development of these products. One of the latest researches used a Kano model to understand food oral processing of confectionery products [111], starting from a field survey on consumer preferences towards oral processing.

## 8. Modelling the Whole Chain

A final class of models attempts to deal with the whole chain at the same time, exploiting a more high-level perspective on the necessary steps. While this is still a relatively unexplored domain, two examples come from lean manufacturing in the confectionary industry, and process simulation for the production of bioethanol.

Tackling the processing step as a whole can provide a high-level evaluation of production, and inform stakeholders on the possible improvements to be performed to optimize their factories. For example, lean manufacturing effects in a confectionery company producing biscuits is presented in [112]. The objective of these researches was to evaluate the effects of implementing lean manufacturing in a Serbian confectionery production company during a period of 24 months. Lean manufacturing tools implemented in the production processes were visual control and single minute exchange of dies (SMED) while maintenance implemented 5S (a workplace organization method based on Sort, Set in order, Shine, Standardize, and Sustain) in combination with total productive maintenance (TPM). Problem solving sessions were tools implemented in these two processes. Results from this research showed that the visual control tables initiated 61 improvement memos out of which 39% were fully implemented. A total of 2284 minor problems were recorded, with over 95% of issues revealed in due time; total SMED time decreased by 7.6%; 19 problem solving sessions were initiated, with 58% of solving effectiveness, and the remaining issues converted to on-going projects. In maintenance, the level of implementation of 5S improved from 29.9 to 60.3, and the overall equipment effectiveness (OEE) indicator increased from 87.9% to 92.3% while mean time between failures (MTBF) increased by 16.4% [112,113].

In another example, *process simulation* is the methodology involving the development of a flowsheet which depicts the sequence of all operating steps and their connecting mass and energy streams present in the integrated process. Conceptual process design refers to the development of a flowsheet for a yet unrealized process given specific raw materials and target final products. *Process synthesis* aims at identifying the best possible flowsheet for a desired transformation, and it is a procedure originally designed for the chemical and petrochemical industry [114].

Nowadays, several companies in domains such as biotech, pharmaceutics, and food production have resorted to adopting process simulation, leading to a proliferation of software tools. Such tools include Aspen Plus and Aspen HYSYS from Aspen Technology (Burlington, MA, USA), ChemCAD from Chemstations (Houston, TX, USA), PRO/II from SimSci (Lake Forest, CA, USA), SuperPro Designer from Intelligen, Inc. (Scotch Plains, NJ, USA), and the ProSim Suite from ProSim (Labège, France).

In the cereal chain in particular, process simulation has been adopted at first for classical products, such as corn starch [115]. More recently, however, motivated by the needs of sustainability and in the context of circular economy, a considerable amount of research and industrial effort has been devoted to the design and operation of cereal biorefineries producing biofuels, bioenergy and valuable chemicals, either directly from cereal grains or from bio-products derived by their harvesting or processing. For biorefineries to be competitive, they have adopted the same process integration and flexible approaches used in the design of oil refineries [116]. Process simulation can play an important role in this effort as a tool to evaluate alternatives, develop integration strategies and eventually optimize plant performance.

Bio-ethanol is the most common product of cereal biorefineries for which process simulation is used. However, the raw material used or the side by-products may differ. Reference [117] used simulation to model bio-ethanol production from corn using the dry-grind process while reference [118] compared alternative technologies using corn stover as raw material. Reference [116] investigated the feasibility of arabinoxylan production in a wheat biorefinery. Processes that valorize wastes from cereal processing have also been simulated. A techno-economic feasibility study was conducted by [119] for the production of succinic acid by fermenting bakery wastes. Reference [120] investigated the production of astaxanthin from agro-industrial wastes including wheat bran.

The need for adopting process simulation tools is expected to become even more pressing in the extended food industry, including cereal processing, because of growing trends such as inventing new processes for valorization of food waste, producing innovative food products, using alternative raw materials, and minimizing environmental impact.

## 9. Conclusions

In this paper, we presented a review of the state-of-the-art for modelling and simulating different steps of the cereal supply chain, from farming to storage, from drying to milling, from processing to consumption.

The models presented in this review, able to deliver simulations and predictions for the outcomes of processing or product qualities, can be roughly divided into two groups: mechanistic, mathematical models that aim to represent the underlying physical realities of the phenomena under study; and empirical, statistical models that attempt to provide predictions for correlated values, built based on available data and expert knowledge. When available, mechanistic models are probably a better choice, as they are able to provide more precise information on processes and product properties; but in many practical cases, the systems under study are so complex, involving different chemical and physical processes, that building mechanistic models is impractical. In such situations, empirical models represent the only possible approach. It is also important to notice that very often models are extremely specialized, for one specific product or process, and thus the choices available to practitioners are necessarily limited.

While a considerable corpus of knowledge can be found in the specialized literature related to each step, the available models are not designed to be integrated at a larger scale. This lack of integration makes it harder to simulate several steps (or even the entire chain) together, and thus at the moment it is still unpractical to explore complex hypothetical scenarios, such as what could happen during major disruptions of the supply chain. Even approaches that try to take into account the whole chain, such as in the examples with confectionary and bioethanol, often adopt such a high-level view of the processing that the detailed repercussions cannot be foreseen.

Furthermore, and perhaps unsurprisingly, the majority of applications are focused on the most consumed cereals and corresponding products: maize (corn), wheat, and rice, that alone contribute to more than half of all calories consumed by human beings [121], and a relevant part of the feed for cattle and other livestock, thus also supporting indirectly human nutrition. Research on modelling other cereals’ supply chains, like barley, sorghum, and millet, is comparatively scarce. More research on these less consumed cereals might lead to richer and more varied diets, and help sustain a growing population.

In conclusion, a more holistic approach to the cereal chain could be beneficial for overcoming the future challenges that could potentially disrupt the supply chain, such as exceptional weather brought by climate change, or pandemics such as the current COVID-19 crisis. The availability of models encompassing the whole supply chain would make it possible to simulate alternative scenarios, allowing companies to both reduce costs and contain their emissions, highlighting issues and opportunities that would otherwise be invisible when analyzing a single step in isolation.

## Data Availability

Not applicable.
